# Sex Differences in Rhythmic Preferences in the Budgerigar (*Melopsittacus undulatus*): A Comparative Study with Humans

**DOI:** 10.3389/fpsyg.2016.01543

**Published:** 2016-10-04

**Authors:** Marisa Hoeschele, Daniel L. Bowling

**Affiliations:** Department of Cognitive Biology, University of ViennaVienna, Austria

**Keywords:** rhythm, acoustic preference, human, budgerigar, music, auditory perception

## Abstract

A variety of parrot species have recently gained attention as members of a small group of non-human animals that are capable of coordinating their movements in time with a rhythmic pulse. This capacity is highly developed in humans, who display unparalleled sensitivity to musical beats and appear to prefer rhythmically organized sounds in their music. Do parrots also exhibit a preference for rhythmic over arrhythmic sounds? Here, we presented humans and budgerigars (*Melopsittacus undulatus*) – a small parrot species that have been shown to be able to align movements with a beat – with rhythmic and arrhythmic sound patterns in an acoustic place preference paradigm. Both species were allowed to explore an environment for 5 min. We quantified how much time they spent in proximity to rhythmic vs. arrhythmic stimuli. The results show that humans spent more time with rhythmic stimuli, and also preferred rhythmic stimuli when directly asked in a post-test survey. Budgerigars did not show any such overall preferences. However, further examination of the budgerigar results showed an effect of sex, such that male budgerigars spent more time with arrthymic stimuli, and female budgerigars spent more time with rhythmic stimuli. Our results support the idea that rhythmic information is interesting to budgerigars. We suggest that future investigations into the temporal characteristics of naturalistic social behaviors in budgerigars, such as courtship vocalizations and head-bobbing displays, may help explain the sex difference we observed.

## Introduction

Although we usually think about rhythm in the context of music, repetitive temporal patterns of acoustic events can be found throughout the animal kingdom, with familiar examples coming from the stridulations of insects, as well as the vocalizations of frogs, birds, and mammals (Wells, 1977; Haimoff, 1986; Geissmann, 2000; Greenfield, 2005; Mann et al., 2006; Hall, 2009). Many species appear specialized for rhythmic sound production, engaging in highly coordinated forms of inter-individual temporal coordination like synchrony and antiphony (Ravignani et al., 2014). In some species, there is also evidence of finely tuned sensitivity to specific temporal patterns. Coo production in Collared doves (*Streptopelia decaocto*), for example, is highly stereotyped in time (Ballintijn and ten Cate, 1999) and conspecifics only respond if coos strictly adhere to this form (Slabbekoorn and ten Cate, 1999; see also Doherty and Hoy, 1985; Gerhardt, 1988).

Despite widespread sensitivity to temporal patterns in the animal kingdom, only a small number of the animals tested so far appear to be able to coordinate their movements in time with a rhythmic pulse, an ability we will refer to as beat perception and motor coordination, or BPMC (Large, 2000; Patel et al., 2005; Repp, 2005; Patel, 2006; Fitch, 2013). Humans all around the world regularly engage in BPMC in response to rhythmic stimuli, often spontaneously, effortlessly and with pleasure (McNeill, 1995; Patel et al., 2005; Janata et al., 2012). Some form of BPMC has been found in every musical tradition where it has been studied (Nettl, 2000), and infant electroencephalographic and looking-preference studies suggest that our sensitivity to the connection between music and movement develops very early in life (∼7 months; Phillips-Silver and Trainor, 2005). This evidence suggest that, for humans, there is something inherently rewarding about moving our bodies in time to music.

Outside of our species, the group best known for clear examples of BPMC is parrots (order Psittaciformes). The first animal in which a capacity for BPMC was conclusively demonstrated was Snowball the sulfur-crested cockatoo (*Cacatua galerita*). Snowball was discovered through a YouTube video posted in 2007 in which he appeared to be timing his movements to a musical beat. Detailed temporal analyses of Snowball’s dancing behavior showed that he intermittently engaged in sequences of between 12 and 36 head-bobs closely aligned with the beat (average phase relation = 3.9°; Patel et al., 2009). This behavior was observed across a range of tempos, and demonstrated to be more likely than expected by chance using simulations. Shortly after Snowball, a temporal analysis of “dancing” animal videos on youtube showed that the majority in which BPMC was statistically supported were in fact of parrots (Schachner et al., 2009). These results suggest that, like humans, parrots may also be motivated to pay special attention to and/or move in time with rhythmic acoustic patterns. An appropriate note of caution here is that it is unknown whether Snowball, or any of the other youtube parrots, were explicitly trained to dance by humans. What can be said, however, is that comparable human-pet interactions involving other species such as cats and dogs have so far failed to produce any similar evidence for BPCM (Schachner et al., 2009). This suggests that BPMC behavior may come more naturally to parrots, even if other many other animals, such as dogs, could be trained to perform BPMC.

One hypothesis that might explain why BPMC occurs in humans and parrots is that both species are vocal learners, that is, one of the few types of animals that learn their vocalizations from exposure to vocalizing conspecifics (Tyack, 2008). The “vocal learning and rhythmic synchronization” hypothesis suggests that the strong neural connectivity between auditory and motor regions required for vocal learning is a prerequisite for BPMC (Patel, 2006). In its strongest formulation, this hypothesis is not supported by data. Despite the fact that many of the species in which there is evidence for BPMC are vocal learners (e.g., humans, several parrots species, and elephants), there are exceptions. Ronan, California sea lion (*Zalophus californicus*), was trained to bob her head in time with musical beats at different tempos despite not being a vocal learner (Cook et al., 2013). Another example is Ai, who is a chimpanzee (*Pan troglodytes*) and thus not a vocal learner. After being trained to tap her finger, Ai showed sequences of BPMC when presented with a repeating acoustic stimulus in the background during tapping (Hattori et al., 2013), as long as its rate was similar to her natural tapping rate (Hattori et al., 2015). Nevertheless, it remains possible that a vocal learning capacity makes BPMC more likely or more intrinsically motivating, making a weaker formulation of the vocal learning and rhythmic synchronization hypothesis plausible.

Regardless whether there is a relationship between BPMC and vocal learning, it is clear that BPMC warrants further investigation in parrots. In humans, the experience of moving to a beat is often considered pleasurable and acoustic stimuli related to wanting to perform BPMC also induce positive affect (Janata et al., 2012). Here, we explore the possibility that budgerigars (*Melopsittacus undulatus*), like humans, find rhythmic temporal patterns to be rewarding. Most existing studies of acoustic preferences have used a place preference paradigm, in which animals can choose to spatially associate with different kinds of sounds. Typically, the animal is allowed to freely move around a space with different sounds playing in different locations (Hoeschele et al., 2015). Such laboratory studies are intended to be analogous to field studies where animals are free to move toward appetitive stimuli played through a speaker. In their study of conspecific song preferences, Leitão et al. (2006) showed that the results of place preference experiments in the lab closely match those found in the field, providing support for the validity of the place preference paradigm. Similarly, Gentner and Hulse (2000) used a place preference paradigm to provide direct evidence that female European starlings (*Sturnus vulgaris*) prefer longer over shorter male song bouts, for which there was previously only correlational evidence. In studies of music-related preferences, place preference experiments have been used to show that newly hatched chicks (*Gallus gallus*) and humans, but not cotton-top tamarins (*Saguinus oedipus*) prefer to associate with melodies composed of consonant as opposed to dissonant tonal relations (McDermott and Hauser, 2004; Chiandetti and Vallortigara, 2011). Further musical place preference studies suggest that cotton top tamarins prefer to listen to silence over music (McDermott and Hauser, 2007), whereas chimpanzees prefer music over silence (Mingle et al., 2014). As far as we know, no studies have yet examined rhythmic preferences in non-human animals.

Accordingly, in this study we tested whether humans and budgerigars exhibit preferences for rhythmic vs. arrhythmic acoustic temporal patterns using a place preference paradigm similar to those in the studies described above. Budgerigars are a small Australian parrot species capable of engaging in BPMC (Hasegawa et al., 2011). Rhythmic patterns were represented by a repeating 2-bar stimulus comprised of percussion instruments. Arrhythmic patterns were represented by the same percussive instruments presented equally often but separated by random temporal intervals. Similar to the studies on consonance/dissonance (McDermott and Hauser, 2004, 2005; Chiandetti and Vallortigara, 2011), we used a paradigm with constant sound playback and a brief exposure period (5 min) to avoid habituation (see Dobson, 1973). We expected humans to spatially associate with the rhythmic pattern. If budgerigars showed similar behavior, it would provide evidence in support of the possibility that rhythmic patterns are attractive and biologically relevant in this species.

## General Materials And Methods

The testing chambers and procedure were matched across experiments. As such, we provide a general description here.

### Ethical Statement

All procedures performed in studies involving human participants were approved by the University of Vienna Ethics Committee (Approval Number 00063) and were conducted in line with the Declaration of Helsinki (1964). All procedures performed in studies involving animals were in accordance with Austrian animal protection and housing laws and were approved by the ethical board of the behavioral research group in the faculty of Life Sciences at the University of Vienna (Approval Number 2015-005).

### Apparatus

**FIGURE 1:**
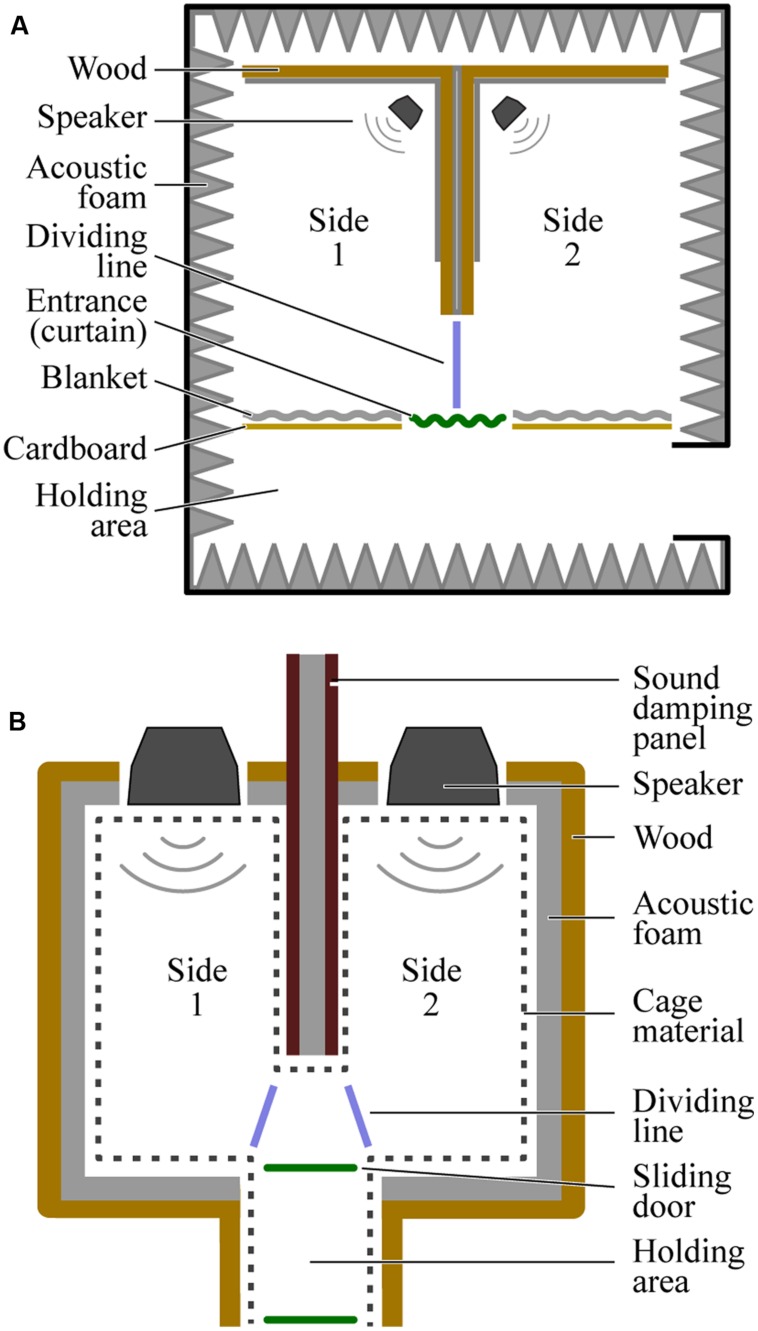
**Overhead views of the testing chambers used for humans **(A)** and budgerigars **(B)**.** The dividing lines (blue) mark the cutoffs used when coding the video data to determine the participants’ location at any given time.

Diagrams of the place preference test chambers used to test each species are provided in **Figure 1**. The chamber for humans and the chamber for budgerigars differed in size but were otherwise similar. In both cases, a large rectangular space was divided in two by a bisecting wall with an opening at one end that provided access between the left and right sides. Critically, the left and right sides were identical: each was empty except for an overhead lighting fixture and a single speaker (M-Audio AV 40, Cumberland, RI, USA) placed at the end opposite the entrance to the chamber. The entrance to both sides was in the middle of one of the long sides of the rectangle, directly perpendicular to the open end of the bisecting wall, such that participants were immediately faced with a choice to go left or right upon entering.

The human chamber was built inside an anechoic room to reduce the transmission of sound from one side of the chamber to the other. The chamber measured 3.5 m (width) × 2.1 m (length) × 3.2 m (height). The exterior walls, floor, and ceiling of the chamber were the walls of the anechoic room. The exception to this was the entrance wall, which was constructed out of large sheets of cardboard and heavy blankets. The bisecting wall was constructed out of heavy sheets of wood, cardboard, and blankets. A curtain was hung over the entrance itself to block visual access between the room and the holding area (3.5 m × 1.2 m × 3.2 m), where participants waited before starting the experiment.

The budgerigar chamber was essentially a smaller version of the human chamber (measuring 0.6 m × 0.5 m × 0.6 m). The outer walls and ceiling were made of wood, covered in acoustic foam on the inside to reduce reflections. To prevent budgerigars’ from chewing on/eating the foam, we installed wire cage material approximately 1 cm in front of the foam. Because the distance between the left and right sides of this chamber was considerably less than that in the human chamber, it was necessary to construct the budgerigar bisecting wall out of specially designed sound absorbing material (Wolf PhoneStar Tri sound dampening plates; Heilsbronn, Germany). Two such plates, separated by acoustic foam were used. The floor of the apparatus was made of a thin layer of wood placed on top of another sound dampening plate. A small holding area (0.15 m × 0.20 m × 0.60 m) attached to the entrance with sliding doors on either side allowed us to place the bird inside and then release it into the chamber at the start of the experiment.

### Stimuli

**FIGURE 2:**
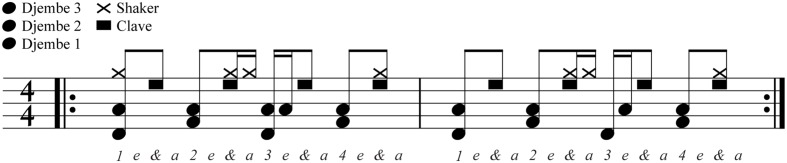
**The composition of instrument events in the rhythmic stimulus in percussion notation.** The full pattern is 3 s, and thus repeated approximately 100 times during the course of the 5 min experiments. Arrhythmic stimuli were constructed by shifting each note randomly for each repetition of the stimulus by ±200 ms (Experiments 1 and 3) or ±50 ms (Experiment 2).

Three sound stimuli were used in this experiment. The *rhythmic* stimulus (**Figure 2**) was a repeating 2-bar pattern of five percussion instruments (3 djembe drums, a clave, and a shaker) recorded from samples in Logic Pro (version 9; Apple, Cupertino, CA, USA). The most energetic frequencies in the samples were approximately 102, 475, 741, 2324 Hz, and between 2800 and 8000 Hz for the drums, clave and shaker respectively. All frequencies were within the audible range for both humans and budgerigars at the amplitudes used in the experiment (75 dB for humans, and 80 dB for budgerigars, see, e.g., Dooling and Saunders, 1975; Okanoya and Dooling, 1987). The two lowest frequency drum samples (djembe drums 1 and 2) occurred alternately every 375 ms suggesting a tempo of 160 beats per minute (BPM). The *arrhythmic ± 200* stimulus had the same underlying pattern as the rhythmic stimulus, but each event was shifted in time by an interval randomly selected from a uniform distribution ranging from -200 to +200 ms, resulting in total disruption of any temporal regularity. Finally, a third stimulus, *arrhythmic ± 50*, was created by changing the uniform distribution to range from -50 and +50 ms, resulting in less disruption of temporal regularity. Instrument samples were assembled into the patterns using custom Matlab (R2013a; Mathworks, Nantick, MA, USA) code. For each experiment, the two stimuli to be contrasted were saved as the left and right tracks of a two-track wav file for presentation (sampling rate = 44100 Hz; bit depth = 32; side counterbalanced across participants). This allowed the sounds on both sides of the test chambers to be controlled from a single file.

### Procedure

The experimental procedure can be described in three steps. First, the experiment was initiated by the experimenter starting playback of the wav file. Second, the participants were permitted to enter the chamber. Third, after 5 min, playback was ended and the participant was released. While inside the chamber, the spatial behavior of each participant was recorded with overhead cameras (two C920 HD Pro Webcam for humans; Logitech, Lausanne, Switzerland; and a single Hero 3+ camera for budgerigars; GoPro, Mountain View, CA, USA). The video data were blind-coded to calculate the proportion of time participants spent on each side of the experimental chamber, which was used as a measure of the participant’s preference for the corresponding stimulus (see below).

## Experiment 1: Humans, Rhythmic vs. Arrhythmic ±200

Our goal in this experiment was to establish that humans show a preference for rhythmic over arrhythmic stimuli using a place preference paradigm designed to be directly comparable with the paradigm used with budgerigars (Experiment 3).

### Materials and Methods

#### Participants

Twenty-five adult humans participated in the experiment (12 males, 13 females) at the University of Vienna (ages: 19–41). They were recruited either directly by a research assistant, or through an online service (Sona Systems; Tallinn, Estonia) where potential participants were registered and could sign up for experiments for monetary compensation. The majority of participants were students at the University of Vienna. None of the participants had any prior knowledge about the experiment. All participants provided informed consent before participation.

#### Stimuli

In this experiment, all participants were presented with the rhythmic stimulus from one speaker, and the arrhythmic ±200 from the other speaker, side counterbalanced across participants.

#### Procedure

Upon arrival at the laboratory, participants were told that the experiment consisted of entering a testing chamber with sounds being played at a comfortable level (75 dB directly in front of each speaker). They were told that they could enter the chamber through the entrance curtain as soon as they heard sound playing inside and that they were free to explore the space inside as they pleased. Lastly, they were told that when the sound ended they could come out of the chamber. Participants then filled out an informed consent form which included the provision that they could withdrawal from the study at any time without further consequences. Once the participant was ready, the experimenter initiated video recording and acoustic playback (both controlled by the same computer; Mac Mini; Apple, Cupertino, CA, USA) and the participant entered the chamber. After 5 min had passed, video recording and stimulus playback were terminated. Following completion of their time in the chamber, participants completed a brief computerized survey (LiveCode Community 7.0.5; Edinburgh, Scotland) in which they listened to each stimulus again and answered the question “how much did you like this sound?” Responses were collected using a continuous scale from 0 (“not at all”) to 100 (“very much”).

#### Video Coding

Participant location for the first 5 min after they had entered the testing chamber was coded by a human observer who was blind to which stimulus was presented on which side. Human participants were coded as being on either side of the chamber if both feet were fully on one side of the bisecting wall (see dividing line, **Figure 1A**). In all other cases, location was coded as being on neither side. We excluded the time that participants were on neither side and calculated the proportion of time spent on the rhythmic side by dividing the time spent on the rhythmic side by the total amount of time spent on both sides.

### Results

**FIGURE 3:**
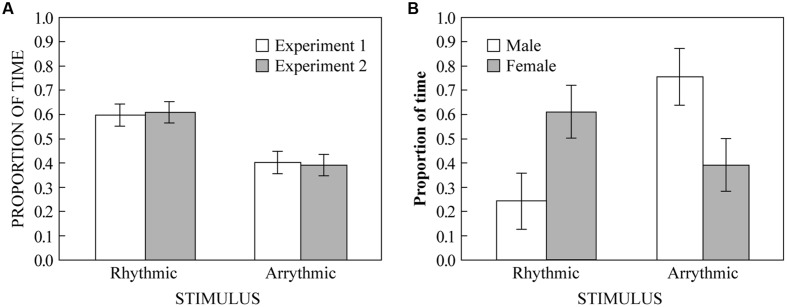
**(A)** The proportion of time humans spent on either side of the apparatus. White bars show results from Experiment 1 (rhythmic vs. arrhythmic ±200 ms). Gray bars show results from Experiment 2 (rhythmic vs. arrhythmic ±50 ms). **(B)** The proportion of time budgerigars spent on either side of the apparatus in Experiment 3, separated by sex bars (white = male; gray = female). Error bars represent ± 1 standard error of the mean.

Three participants only visited one side (all female). Because they could not have heard both stimuli without being on both sides, and because we were looking for a preference between the two stimuli, we excluded their data from the analysis. We conducted a one-sample two-tailed *t*-test looking whether proportion of time spent on the rhythmic side of the apparatus was different from chance (0.5) across the remaining 22 subjects. We found that participants spent significantly more time on the rhythmic side (*M* = 0.60; *SD* = 0.22) than would be expected by chance [*t*(21) = 2.111, *p* = 0.047]. These results are displayed in **Figure 3A** along with the data from Experiment 2 (see below). The survey data also showed a significant overall preference for the rhythmic stimulus (*M* = 82; *SD* = 11) compared to the arrhythmic [*M* = 42; *SD* = 34; *t*(21) = 5.47, *p* < 0.0001]. Furthermore, the difference between survey responses (rhythmic–arrhythmic) was positively correlated with the proportion of time spent on the rhythmic stimulus side in the place preference experiment (*r* = 0.486, *p* = 0.022), suggesting that spatial behavior in the place preference paradigm is related to subjective preference across individuals.

## Experiment 2: Humans, Rhythmic vs. Arrhythmic ±50

The results of Experiment 1 suggest that humans prefer rhythmic over arrhythmic temporal patterns, However, the arrhythmic stimulus we used was far removed from anything humans might experience in music because the elements were so heavily shifted that the pattern was lost completely. Our goal in Experiment 2 was to determine whether the results of Experiment 1 hold using a less temporally disturbed stimulus. This experiment was thus exactly the same as Experiment 1, except that arrhythmic ±200 stimulus was replaced by arrhythmic ±50.

### Materials and Methods

#### Participants

None of the participants from Experiment 1 participated in Experiment 2. All participants were naïve to the experimental setup. Twenty adult humans participated in the experiment (10 males, 10 females) at the University of Vienna (age: 20–30). They were recruited in the same manner as in Experiment 1.

### Stimuli, Procedure, and Coding

In this experiment, all participants were presented with the rhythmic stimulus from one speaker, and the arrhythmic ±50 from the other speaker, side counterbalanced across participants. The procedure and video coding were conducted in the same manner as Experiment 1.

### Results

Two participants only visited one side (one male, one female). As in Experiment 1, we excluded their data from the analysis. We conducted a one-sample two-tailed *t*-test looking at whether the proportion of time spent on the rhythmic side of the apparatus was significantly different from chance (0.5) across the remaining 18 subjects. We found that participants spent significantly more time on the rhythmic side (*M* = 0.61; *SD* = 0.19) than would be expected by chance [*t*(17) = 2.490, *p* = 0.023]. These results are displayed in **Figure 3A** along with the data from Experiment 1. The survey data also showed a significant overall preference for the rhythmic stimulus (*M* = 65; *SD* = 25) compared to the arrhythmic [*M* = 29; *SD* = 23; *t*(17) = 4.22, *p* = 0.001]. However, unlike in Experiment 1, the difference between survey responses (rhythmic–arrhythmic) was not significantly correlated with the proportion of time spent on the rhythmic stimulus side in the place preference experiment (*r* = -0.097, *p* = 0.700).

## Experiment 3: Budgerigars, Rhythmic vs. Arrhythmic ±200

Because we were able to replicate our result with humans and show that they consistently spent more time with rhythmic than arrhythmic stimuli in our place preference chamber, we decided to use a smaller but otherwise similar chamber to test budgerigars using the same stimuli we had tested with humans in Experiment 1.

### Materials and Methods

#### Participants

Sixteen budgerigars participated in the task (eight males, eight females). All birds were naïve to the test chamber and the stimuli. When not in the experimental chamber, these birds are housed together in mixed-sex groups of eight in two separate aviaries (2 m × 1 m × 2 m) within the same room.

### Stimuli

In this experiment, all birds were presented with the rhythmic stimulus from one speaker, and the arrhythmic ±200 from the other speaker, side counterbalanced across participants.

#### Procedure

All birds in our study were first habituated to the test chamber. We habituated birds so that only the stimuli presented, not the environment itself, would be novel during testing to increase the chance that any difference in time spent on either side was due to the sound. All birds were placed in the test chamber for 5 min sessions at least six times to allow adjustment to the test chamber. Birds continued these habituation sessions until they had explored both sides on at least three sessions. These criteria were intended to increase the chance that the budgerigars would fully explore the test chamber and thus be exposed to both sounds during testing. Habituation sessions were conducted like rhythm testing sessions (explained below) except that the rhythmic and arrhythmic stimuli were replaced with either budgerigar sounds (sampled from the CD album “Budgerigar Country”; Skeotch and Koschak, 2010) or silence.

After habituation, we tested the birds with the same rhythmic and arrhythmic stimuli that we had presented to the humans in Experiment 1. Birds were individually placed in the holding area. The experimenter then initiated video recording and acoustic playback and subsequently opened the sliding door that allowed access from the holding area into the test chamber. This sliding door was closed as soon as budgerigars had entered the apparatus. The holding area was kept dark, whereas there was light on both sides of the test chamber. This was intended to encourage the birds to leave the holding area quickly after the door was opened. As in Experiment 1, 5 min after the bird had entered the chamber, video recording and stimulus playback were terminated and the bird was taken back to its home aviary.

#### Video Coding

Video coding was conducted in a similar way to Experiments 1 and 2. However, because the bisecting wall separating the two rooms in the budgerigar test chamber was considerably larger than the budgerigars themselves, and because budgerigars’ ears are not directly above their legs (as in humans), we coded budgerigars as being on a particular side of the apparatus when their entire head had passed over either of the dividing lines shown in **Figure 1B**. When their head was between the two dividing lines, they were coded as being on neither side. We excluded the time that budgerigars spent on neither side and calculated the proportion of time spent on each side by dividing the time spent on one side by the total amount of time spent on both sides.

### Results

Four budgerigars only visited one side (three males, one female). Following the same procedure as Experiments 1 and 2 (for the same reasons), we excluded their data from the analysis. For the remaining 12 budgerigars, we conducted a one-sample two-tailed *t*-test looking whether the proportion of time spent on the rhythmic side was different from chance (0.5) across subjects. We found no significant difference between the amount of time spent on the rhythmic side of the test chamber and chance [*t*(11) = 0.289, *p* = 0.778]. However, when looking at the raw data, we noticed that male birds appeared to perform differently than female birds, we thus compared the proportion of time spent on the rhythmic side between male and female birds using a Welch’s *t*-test because of differences in sample sizes between males and females. We found a significant difference between the amount of time spent by male and female birds on the rhythmic side [*t*(10) = 2.410, *p* = 0.038] such that male birds spent less time on the rhythmic side (*M* = 0.25, *SD* = 0.26) and female birds spent more time on the rhythmic side (*M* = 0.61, *SD* = 0.29). These results are displayed in **Figure 3B**.

## Discussion

The results show that humans not only prefer rhythmic over arrhythmic stimuli, they also spend more time with rhythmic stimuli than arrhythmic stimuli in an acoustic place preference paradigm. This result was replicable even when we adjusted our arrhythmic stimulus to deviate less from the rhythmic stimulus. When we studied budgerigars using highly similar methods, we found that there was no overall preference for rhythmic or arrhythmic stimuli. However, this appears to have been due to sex-dependent differences in the behavior of these birds: males spent more time with arrhythmic stimuli and females spent more time with rhythmic stimuli.

Our results support the idea that rhythmic information is interesting to budgerigars. Although we cannot determine the cause of the sex difference we found, one possibility is that it is related to sexual dimorphism in courtship behaviors and vocalizations. Similar to many songbird species, male budgerigars sing to attract females. Their “warble song” is acoustically distinct from other budgerigar vocalizations (produced by both sexes) and is also perceived as distinct by other budgerigars (Tu et al., 2011). The warble song is also often accompanied by a repetitive head bobbing display, sometimes followed by bill touching, especially in bonded pairs (Zocchi and Brauth, 1991). To a human observer, male head bobbing resembles the head bobbing observed in other parrot species engaged in BPS. Female sensitivity to male displays is common in birds (e.g., Searcy and Marler, 1981; Borgia, 1995; Forstmeier et al., 2002; Ballentine et al., 2004; Amy et al., 2008; Hoeschele et al., 2010). Thus, it is possible that the apparently repetitive form of male budgerigar displays may underlie the female preference for rhythmic patterns observed in the present experiment. However, we emphasize that the temporal characteristics of the head bobbing display, its relation to the warble song, and female mate choice have not been characterized. The possibility that these factors are related to female preference behavior in the present study is thus necessarily speculative at present.

Additional caution in interpreting the results Experiment 3 is advised by previous findings on how budgerigars perceive and respond to rhythmic stimuli. While budgerigars have been shown to be able to entrain to a beat (Hasegawa et al., 2011), other work suggests that they do not perceive temporal patterns in the same way humans do. Specifically, budgerigars appear to primarily attend local features (such as the absolute length of silence between a specific pair of notes) and ignore global features (such as the relative length of time between all notes being equal) when trained to discriminate between regular and irregular temporal patterns (ten Cate et al., 2016). Similar results have been found in studies with pigeons (Hagmann and Cook, 2010) and zebra finches (van der Aa et al., 2015; ten Cate et al., 2016). However, this seemingly avian lack of attention to global features is not present in all individuals (ten Cate et al., 2016) and may be less pronounced in other species (e.g., starlings; Hulse et al., 1984; jackdaws, *Corvus monedula*; Reinert, 1965). Nevertheless, taken together these results suggest that overall temporal regularity may not have been the feature that attracted female budgerigars to spend more time with the rhythmic stimulus. A possible alternative is that females were interested in hearing the individual drum samples, some of which tended to overlap less in time in the rhythmic compared to arrhythmic stimuli and may have thus been easier to resolve. However, given that very few budgerigars have been tested on their perception of rhythm, and that methodologies differ across the bird species tested so far, it is difficult to draw broad conclusions about budgerigars, parrots, vocal learners, or indeed birds in general. What does seem clear though is that the perception of rhythm in birds is different from that of humans and requires further exploration.

We also believe it is important to interpret our results in a broader context. While Experiment 3 was designed to assess acoustic preferences in budgerigars, we need to be careful not to over-interpret the meaning of “preference” in this context. In Experiments 1 and 2, because we were able to directly survey participants, we were able to compare reported preference with behavior in our place preference paradigm. While reported preference was found to be significantly correlated with behavior in Experiment 1, it was not in Experiment 2. However, in Experiment 2 almost every participant spent more time on the rhythmic side (only 2/18 participants did not, whereas 6/22 did not in Experiment 1). In addition, all participants that showed more than an eight point difference in preference between rhythmic and arrhythmic stimuli (on the 100 point rating scale) preferred the rhythmic stimulus. Thus, although individual behavioral and survey data did not correlate with one another, overall humans both spent more time on the rhythmic side and preferred the rhythmic stimulus in both experiments. A correlation would only exist if, on an individual level, the degree of preference in the survey was directly related to the amount of time spent on the rhythmic side. It seems likely that while these measures are related insofar as we found the same preference in both domains, the degree of preference was not parallel on this particular experiment. Finally, we note that the difference between the rhythmic and arrhythmic stimulus in Experiment 2 was far less than in Experiment 1, which implies that the results should not necessarily be expected to be the same. Overall, because there was agreement in the place preference paradigm and the post-test survey, the place preference paradigm appears to be a reasonable method to assess acoustic preferences in humans. An alternative interpretation is that, because we surveyed participants after they completed the place preference task, their reported preferences may be explained by a “mere exposure” effect, in which they liked the stimulus they were more familiar with (Zajonc, 1968). We believe this is unlikely, however, because if participants had not had any preference for rhythmic vs. arrhythmic stimuli before participation, we would have expected half of them to spend more time on the rhythmic side and the other on the arrhythmic side, producing corresponding responses on the survey afterward. Also, if fundamental aspects of music found across cultures can be taken to reflect our acoustic preferences, there is considerable additional evidence that humans have a preference for rhythmic patterns (Brown and Jordania, 2013).

Even with reasonable confidence that human behavior in our paradigm reflects preference, this does not imply that the same conclusion holds for other species. In particular, it is difficult to distinguish between a preference for hearing a certain acoustic stimulus, and a functional response toward that stimulus. For example, many studies have shown that female mate choice in birds is based on acoustical information derived from male song (e.g., Searcy and Marler, 1981; Forstmeier et al., 2002; Ballentine et al., 2004; Amy et al., 2008; Hoeschele et al., 2010). Does this mean that females prefer to hear attractive male songs over unattractive male songs? Similar place preference paradigms suggest that they do (e.g., Gentner and Hulse, 2000; Leitão et al., 2006). However, it is possible that these songs are purely indicators of male status and females respond in a functional manner (moving toward the attractive stimulus) to secure an attractive mate. In the end, we believe it is not possible to make this distinction. In the present context, we thus define preference as the tendency to spatially associate with a given stimulus. Further attributions of internal states, such as “liking” or “enjoyment,” may be appropriate, but cannot be justified on the basis of our results.

We based our version of the place preference paradigm on previous work studying music-related preferences in animals (McDermott and Hauser, 2004, 2005; Chiandetti and Vallortigara, 2011). One limitation of this two-sided place preference paradigm used here and in these previous studies is that preference for one acoustic pattern is always confounded with avoidance of the other. Consequently it is not possible to determine whether behavior in this paradigm reflects attraction or repulsion. While both possibilities are consistent with preference in the sense that an animal may be more attracted to, or less repulsed by, a given stimulus, this confound presents further obstacles to claims regarding liking or enjoyment. For these reasons, we are planning to move to the use of a three-sided preference apparatus in future experiments where in addition to associating with either of two stimuli, animals can also choose silence. Additionally, now that a human place preference for rhythmic stimuli has been established, we plan to exercise more flexibility in future studies in designing stimuli for budgerigars, increasing the frequency and tempo of events in auditory patterns to better suit their auditory perceptual abilities (Dooling et al., 2002).

On the whole, the study of rhythm perception in avian species is still in its infancy. Early studies of avian pitch perception also showed that birds primarily paid attention to local features rather than global features in laboratory experiments (e.g., Hulse and Cynx, 1985), which is very similar to recent results on rhythm perception (ten Cate et al., 2016). These early studies on pitch perception made it clear that birds tended to be better at assessing local pitch features than mammals (i.e., absolute pitch, Weisman et al., 2010). However, further studies suggested that birds can also pay attention to more global features depending on the context (Bregman et al., 2012; Hoeschele et al., 2012) and how we as experimenters break down the acoustic signal (Bregman et al., 2016). The same might well turn out to be true for rhythm.

## Author Contributions

MH and DB designed the experiment and built the apparatus together. DB created the stimuli. DB was in charge of running the human participants and MH ran the budgerigars. MH analyzed the data and wrote the first draft of the article. DB contributed to data analysis and both authors worked to revise the article. DB made the figures for the article.

## Conflict of Interest Statement

The authors declare that the research was conducted in the absence of any commercial or financial relationships that could be construed as a potential conflict of interest.

## References

[B1] Amy M. Monbureau M. Durand C. Gomez D. Thery M. Leboucher G. (2008). Female canary mate preferences: differential use of information from two types of male–male interaction. *Anim. Behav.* 76 971–982. 10.1016/j.anbehav.2008.03.023

[B2] Ballentine B. Hyman J. Nowicki S. (2004). Vocal performance influences female response to male bird song: an experimental test. *Behav. Ecol.* 15 163–168. 10.1093/beheco/arg090

[B3] Ballintijn M. R. ten Cate C. (1999). Variation in number of elements in the perch-coo vocalization of the collared dove (*Streptopelia decaocto*) and what it may tell about the sender. *Behaviour* 136 847–864. 10.1163/156853999501603

[B4] Borgia G. (1995). Complex male display and female choice in the spotted bowerbird: specialized funcgtions for different bower decorations. *Anim. Behav.* 49 1291–1301. 10.1006/anbe.1995.0161

[B5] Bregman M. R. Patel A. D. Gentner T. Q. (2012). Stimulus-dependent flexibility in non-human auditory pitch processing. *Cognition* 122 51–60. 10.1016/j.cognition.2011.08.008 21911217 PMC3215778

[B6] Bregman M. R. Patel A. D. Gentner T. Q. (2016). Songbirds use spectral shape, not pitch, for sound pattern recognition. *Proc. Natl. Acad Sci. U.S.A.* 113 1666–1671. 10.1073/pnas.1515380113 26811447 PMC4760803

[B7] Brown S. Jordania J. (2013). Universals in the world’s musics. *Psychol.Music* 41 229–248. 10.1177/0305735611425896

[B8] Chiandetti C. Vallortigara G. (2011). Chicks like consonant music. *Psychol. Sci.* 22 1270–1273. 10.1177/0956797611418244 21934134

[B9] Cook P. Rouse A. Wilson M. Reichmuth C. (2013). A California sea lion (*Zalophus californianus*) can keep the beat: motor entrainment to rhythmic auditory stimuli in a non vocal mimic. *J. Comp. Psychol.* 127 412–427. 10.1037/a0032345 23544769

[B10] Dobson C. W. (1973). Song as a reinforcer in the white-crowned sparrow. *Behav. Biol.* 9 719–729. 10.1016/S0091-6773(73)80132-4 4764253

[B11] Doherty J. A. Hoy R. R. (1985). Communication in insects. III. The auditory behavior of crickets: some views of genetic coupling, song recognition, and predator detection, *Q. Rev. Biol.* 60 457–472. 10.1086/414566

[B12] Dooling R. J. Leek M. R. Gleich O. Dent M. L. (2002). Auditory temporal resolution in birds: discrimination of harmonic complexes. *J. Acoust. Soc. Am.* 112 748–759. 10.1121/1.1494447 12186054

[B13] Dooling R. J. Saunders J. C. (1975). Hearing in the parakeet (*Melopsittacus Undulatus*): absolute thresholds, critical ratios, frequency difference limens, and vocalizations. *J. Comp. Physiol. Psychol.* 88 1–20. 10.1037/h0076226 1120787

[B14] Fitch W. T. (2013). Rhythmic cognition in humans and animals: distinguishing meter and pulse perception. *Front. Syst. Neurosci.* 7:68 10.3389/fnsys.2013.00068 PMC3813894 24198765

[B15] Forstmeier W. Kempenaers B. Meyer A. (2002). A novel song parameter correlates with extra-pair paternity and reflects male longevity. *Proc. Biol.Sci.* 269 1479–1485. 10.1098/rspb.2002.2039 12137578 PMC1691048

[B16] Geissmann T. (2000). “Gibbon songs and human music from an evolutionary perspective,” in *The Origins of Music*, eds Wallin N. L. Merker B. Brown S. (Cambridge, MA: MIT Press), 103–123.

[B17] Gentner T. Q. Hulse S. H. (2000). Female european starling preference and choice for variation in conspecific male song. *Anim. Behav.* 59 443–458. 10.1006/anbe.1999.1313 10675267

[B18] Gerhardt H. C. (1988). “Acoustic properties used in call recognition by frogs and toads,” in *The Evolution of the Amphibian Auditory System*, eds Fritzsch B. Hethington T. Ryan M. J. Wilczynski W. Walkowiak W. (New York, NY: Wiley), 21:455-483.

[B19] Greenfield M. D. (2005). Mechanisms and evolution of communal sexual displays in arthropods and anurans. *Adv. Stud. Behav.* 35 1–62. 10.1016/S0065-3454(05)35001-7

[B20] Hagmann C. E. Cook R. G. (2010). Testing meter, rhythm, and tempo discriminations in pigeons. *Behav. Process* 85 99–110. 10.1016/j.beproc.2010.06.015 20600695

[B21] Haimoff E. H. (1986). Convergence in the duetting of monogamous old world primates. *J. Hum. Evol.* 15 51–59. 10.1016/S0047-2484(86)80065-3?

[B22] Hall M. L. (2009). A review of vocal duetting in birds. *Adv. Stud. Behav.* 40 67–121. 10.1016/S0065-3454(09)40003-2

[B23] Hasegawa A. Okanoya K. Hasegawa T. Seki Y. (2011). Rhythmic synchronization tapping to an audio-visual metronome in budgerigars. *Sci. Rep.* 1:120 10.1038/srep00120 PMC3216601 22355637

[B24] Hattori Y. Tomonaga M. Matsuzawa T. (2013). Spontaneous synchronized tapping to an auditory rhythm in a chimpanzee. *Sci. Rep.* 3:1566 10.1038/srep01566 PMC3610097 23535698

[B25] Hattori Y. Tomonaga M. Matsuzawa T. (2015). Distractor effect of auditory rhythms on Self- Paced Tapping in Chimpanzees and Humans. *PLoS ONE* 10:e0130682 10.1371/journal.pone.0130682 PMC4488575 26132703

[B26] Hoeschele M. Guillette L. M. Sturdy C. B. (2012). Biological relevance of acoustic signal affects discrimination performance in a songbird. *Anim. Cogn.* 15 677–688. 10.1007/s10071-012-0496-8 22526691

[B27] Hoeschele M. Merchant H. Kikuchi Y. Hattori Y. ten Cate C. Hoeschele M. (2015). Searching for the origins of musicality across species. *Philos. Trans. R Soc. B* 370:20140094 10.1098/rstb.2014.0094 PMC4321135 25646517

[B28] Hoeschele M. Moscicki M. K. Otter K. A. van Oort H. Fort K. T. Farrell T. M. (2010). Dominance signalled in an acoustic ornament. *Anim. Behav.* 79 657–664. 10.1016/j.anbehav.2009.12.015

[B29] Hulse S. H. Cynx J. (1985). Relative pitch perception is constrained by absolute pitch in songbirds (*Mimus*, *Molothrus*, and *Sturnus*). *J. Comp. Psychol.* 99 176–196. 10.1037//0735-7036.99.2.176

[B30] Hulse S. H. Humpal J. Cynx J. (1984). Discrimination and generalization of rhythmic and arrhythmic sound patterns by european starlings (*Sturnus vulgaris*), *Music Percept*. 1 442–464. 10.2307/40285272

[B31] Janata P. Tomic S. T. Haberman J. M. (2012). Sensorimotor coupling in music and the psychology of the groove. *J. Exp. Psychol. Gen.* 141 54–75. 10.1037/a0024208 21767048

[B32] Large E. W. (2000). On synchronizing movements to music. *Hum. Mov. Sci.* 19 527–566. 10.1016/S0167-9457(00)00026-9

[B33] Leitão A. ten Cate C. Riebel K. (2006). Within-song complexity in a songbird is meaningful to both male and female receivers. *Anim. Behav.* 71 1289–1296. 10.1016/j.anbehav.2005.08.008

[B34] Mann N. I. Dingess K. A. Slater P. (2006). Antiphonal four-part synchronized chorusing in a neotropical wren. *Biol. Lett.* 2 1–4. 10.1098/rsbl.2005.0373 17148310 PMC1617190

[B35] McDermott J. Hauser M. D. (2004). Are consonant intervals music to their ears? Spontaneous acoustic preferences in a nonhuman primate. *Cognition* 94 B11–B21. 10.1016/j.cognition.2004.04.004 15582619

[B36] McDermott J. Hauser M. D. (2005). Probing the evolutionary origins of music perception. *Ann. N. Y. Acad. Sci.* 1060 6–16. 10.119/annals.1360.002 16597745

[B37] McDermott J. Hauser M. D. (2007). Nonhuman primates prefer slow tempos but dislike music overall. *Cognition* 104 654–668. 10.1016/j.cognition.2006.07.011 16935277

[B38] McNeill H. W. (1995). *Keeping Together in Time: Dance and Drill in Human History*. Cambridge, MA: Harvard University Press.

[B39] Mingle M. E. Eppley T. M. Campbell M. W. Hall K. Horner V. de Waal F. B. M. (2014). Chimpanzees prefer African and Indian music over silence. *J. Exp. Psychol. Anim. Behav. Process* 40 502–505. 10.1037/xan0000032 PMC4461656 25546107

[B40] Nettl B. (2000). “An ethnomusicologist contemplates universal in musical sound and musical culture,” in The origins of music, eds Wallin N. L. Merker B. Brown S. (Cambridge, MA: MIT Press), 463–472.

[B41] Okanoya K. Dooling R. J. (1987). Hearing in passerine and psittacine birds: a comparative study of absolute and masked auditory thresholds. *J. Comp. Psychol.* 101 7–15. 10.1037/0735-7036.101.1.7 3568610

[B42] Patel A. D. Iversen J. R. Bregman M. R. Schulz I. (2009). Experimental evidence for synchronization to a musical beat in a nonhuman animal. *Curr. Biol.* 19 827–830. 10.1016/j.cub.2009.03.038 19409790

[B43] Patel A. D. (2006). Musical rhythm, linguistic rhythm, and human evolution. *Music Percept.* 24 99–104. 10.1037/a0032345

[B44] Patel A. D. Iversen J. R. Chen Y. Repp B. H. (2005). The influence of metricality and modality on synchronization with a beat. *Exp. Brain Res.* 163 226–238. 10.1007/s00221-004-2159-8 15654589

[B45] Phillips-Silver J. Trainor L. J. (2005). Feeling the beat: movement influences infant rhythm perception. *Science* 308:1430 10.1126/science.1110922 15933193

[B46] Ravignani A. Bowling D. Fitch T. (2014). Chorusing, synchrony and the evolutionary functions of rhythm. *Front. Psychol.* 5:1118 10.3389/fpsyg.2014.01118 PMC4193405 25346705

[B47] Reinert J. (1965). Takt- und rhythmusunterscheidung bei dohlen. *Z. Tierpsychol.* 22 623–671. 10.1111/j.1439-0310.1965.tb01683.x 5888489

[B48] Repp B. H. (2005). Sensorimotor synchronization: a review of the tapping literature. *Psychon. Bull. Rev.* 12 969–992. 10.3758/BF03206433 16615317

[B49] Schachner A. Brady T. F. Pepperberg I. M. Hauser M. D. (2009). Spontaneous motor entrainment to music in multiple vocal mimicking species. *Curr. Biol.* 19 831–866. 10.1016/j.cub.2009.03.061 19409786

[B50] Searcy W. A. Marler P. A. (1981). A test for responsiveness to song structure and programming in female sparrows. *Science* 213 926–928. 10.1126/science.213.4510.926 17775278

[B51] Skeotch A. Koschak S. (2010). *Budgerigar Country*. Available at: http://www.listeningearth.com/LE/p-13-budgerigar-country

[B52] Slabbekoorn H. ten Cate C. (1999). Collared dove responses to playback: slaves to the rhythm. *Ethology* 105 377–391. 10.1046/j.1439-0310.1999.00420.x

[B53] ten Cate C. Spierings M. Hubert J. Honing H. (2016). Can birds perceive rhythmic patterns? A review and experiments on a songbird and a parrot species. *Front. Psychol.* 7:730 10.3389/fpsyg.2016.00730 PMC4872036 27242635

[B54] Tu H.-W. Osmanski M. S. Dooling R. J. (2011). Learned vocalizations in budgerigars (*Melopsittacus undulatus*): the relationship between contact calls and warble song. *J. Acoust. Soc. Am.* 129 2289–2297. 10.1121/1.3557035 21476684 PMC3087398

[B55] Tyack P. L. (2008). Convergence of calls as animals form social bonds, active compensation for noisy communication channels, and the evolution of vocal learning in mammals. *J. Comp. Psychol*. 122 319–331. 10.1037/a0013087 18729661

[B56] van der Aa J. Honing H. ten Cate C. (2015). The perception of regularity in an isochronous stimulus in zebra finches (*Taeniopygia guttata*) and humans. *Behav. Process* 115 37–45. 10.1016/j.beproc.2015.02.018 25725348

[B57] Weisman R. G. Balkwill L.-L. Hoeschele M. Moscicki M. K. Bloomfield L. L. Sturdy C. B. (2010). Absolute pitch in boreal chickadees and humans: exceptions that test a phylogenetic rule. *Learn. Motiv.* 41 156–173. 10.1016/j.lmot.2010.04.002

[B58] Wells K. D. (1977). The social behaviour of anuran amphibians. *Anim. Behav.* 25 666–693. 10.1016/0003-3472(77)90118-X

[B59] Zajonc R. B. (1968). Attitudinal effects of mere exposure. *J. Pers. Soc. Psychol.* 9 1–27. 10.1080/02699931.2010.497409 5667435

[B60] Zocchi D. C. Brauth S. E. (1991). An experimental study of mate directed behaviour in the budgerigar *Melopsittacus undulatus*. *Bird Behav.* 9 49–57.

